# The long non-coding RNA CCAT2 is up-regulated in ovarian cancer and associated with poor prognosis

**DOI:** 10.1186/s13000-016-0499-x

**Published:** 2016-06-10

**Authors:** Shuying Huang, Cheng Qing, Zikun Huang, Yuanfang Zhu

**Affiliations:** Department of Obstetrics and Gynecology, the First Affiliated Hospital of Nanchang University, Nanchang, 330006 Jiangxi China; Intensive Care Unit, the First Affiliated Hospital of Nanchang University, Nanchang, 330006 Jiangxi China; Department of Clinical Laboratory, the First Affiliated Hospital of Nanchang University, Nanchang, 330006 Jiangxi China; Department of Obstetrics and Gynecology, Shenzhen Baoan Matemal and Chlid Health Hospital, Shenzhen, 518133 Guangdong China

**Keywords:** Ovarian cancer, LncRNAs, Colon cancer associated transcript 2, Prognosis

## Abstract

**Background:**

Ovarian cancer is a malignant tumor with a poor prognosis. Accumulating evidence demonstrates that long non-coding RNAs (lncRNAs) are emerging regulators in cancer biology, and can be used as potential biomarkers for cancer diagnosis, prognosis and targeted therapy. The lncRNA CCAT2 (colon cancer associated transcript 2) was recently shown to be involved in several cancers; however, its role in ovarian cancer remains unknown.

**Methods:**

Expression levels of the lncRNA CCAT2 in ovarian cancer tissues, adjacent normal tissues, and cell lines were assessed by quantitative real-time PCR. Then, the associations of CCAT2 expression levels with clinicopathological features and prognosis were evaluated. In addition, CCAT2 functions in tumor progression and invasion were further determined by siRNA-induced CCAT2 silencing *in vitro*.

**Results:**

Expression levels of the lncRNA CCAT2 in ovarian cancer tissues and cell lines were significantly higher compared with values obtained for adjacent non-tumor tissues and normal ovarian epithelial cells. Interestingly, higher CCAT2 expression levels were associated with a shorter overall survival (*P* = 0.006) and disease-free survival (*P* = 0.001) in ovarian cancer patients. In addition, CCAT2 expression was positively correlated with FIGO stage (*P* = 0.002), tumor grade (*P* = 0.006) and distant metastasis (*P* < 0.001). Moreover, CCAT2 knockdown in ovarian cancer cells markedly suppressed cell proliferation, migration, and invasion.

**Conclusions:**

The lncRNA CCAT2 is a novel factor involved in ovarian cancer progression, and constitutes a potential prognostic biomarker and therapeutic target for patients with ovarian cancer.

## Background

Ovarian cancer is the leading cause of death from gynecological malignancies worldwide. More than 230,000 new cases are diagnosed each year, usually at a late stage; this results in the death of about 140,000 women [1]. Over the past decade, a relatively limited improvement of survival rates has been achieved in ovarian cancer. A 5-year survival rate over 90 % can be achieved with patients diagnosed and treated at early stages (I and II). Unfortunately, most ovarian cancer patients are diagnosed with advanced disease (stages III and IV), in which 5-year survival is less than 30 % [2]. Therefore, there is an urgent need to identify new biomarkers and novel therapeutic targets for therapeutic strategy improvement.

Long non-coding RNAs (lncRNAs) are larger than 200 nucleotides; they constitute an emerging class of key regulatory RNAs that do not code for proteins, and are crucial players in numerous biological processes, including chromatin remodeling, gene regulation, dosage compensation, RNA maturation (splicing, editing), and genomic imprinting [3]. Abnormal expression of lncRNAs was reported in different types of cancers [4–8]. In addition, recent studies demonstrated that differential expression of lncRNAs is associated with diagnostic and prognostic markers for ovarian [9], lung [10], gastric [11] and liver [12] cancers. However, the role of lncRNAs in ovarian cancer has only recently been investigated and remains largely unknown.

The lncRNA CCAT2 (colon cancer-associated transcript 2) transcript maps to the highly conserved 8q24.21 region encompassing rs6983267; CCAT2 plays a crucial role in promoting tumor metastasis, growth, and chromosomal instability in colon [13], lung [14], breast [15], and gastric [16] cancers. In colon cancer, CCAT2-enhances invasion and metastasis through the MYC-regulated miRNA-17-5p and miRNA-20a [13]. CCAT2 also appears to be involved in migration and chemoresistance in a SNP-independent fashion in breast cancer [15]. However, CCAT2 expression in ovarian cancer and the underlying mechanism remain unknown.

This study assessed CCAT2 expression levels in ovarian cancer tissues, normal ovarian tissues and cell lines, by quantitative real-time PCR (qRT-PCR). Then, correlation analysis was carried out to establish the associations of CCAT2 expression levels with clinicopathological features and prognosis. Finally, CCAT2 effects on ovarian cancer cell proliferation, migration and invasion were further determined via suppression of the lncRNA CCAT2 *in vitro*.

## Methods

### Patients and samples

A total of 109 ovarian cancer and 45 normal ovarian tissue samples were obtained from patients undergoing wedge biopsy of the ovaries or adnexectomy due to myoma or adenomyosis, in Department of Obstetrics and Gynecology, the First Affiliated Hospital of Nanchang University (Nanchang, China), between 2007 and 2009. No patients had received chemotherapy or radiotherapy prior to surgery. Ovarian cancer was validated by histological examination in all cases according to World Health Organization criteria [17]. Ovarian cancer and normal ovarian tissue specimens excised surgically from patients were immediately snap-frozen and stored in liquid nitrogen until use. Patient characteristics are summarized in Table 1, including age, histological subtype, distant metastasis, tumor size, tumor grade, and FIGO stage (2009). In ovarian cancer, primary cytoreductive surgery followed by platinum-based chemotherapy usually is the mainstay of treatment. All the enrolled women with ovarian cancer in this study received primary cytoreductive surgery followed by platinum-based chemotherapy as follows: paclitaxel (135 mg/m^2^) and cisplatin (75 mg/m^2^) or paclitaxel (175 mg/m^2^) and carboplatin (AUC6), administered every 3 weeks for 6 cycles. Informed consent was obtained from all patients, and the study was approved by the ethics committee of the First Affiliated Hospital of Nanchang University (License No. 2013005).

**Table 1:** Correlation between CCAT2 expression and clinicopathological features in ovarian cancer patients

Parameters	Group	Total	lncRNA CCAT2		*P*-value
			High	Low	
Age (years)	< 55	50	24	26	0.702
	≥ 55	59	31	28	
Histological subtypes	Serous	78	40	38	0.918
	Endometrioid	8	5	3	
	Mucinous	7	3	4	
	Clear cell	9	4	5	
	Others	7	3	4	
FIGO stage	I + II	33	9	24	0.002
	III + IV	76	46	30	
Grade	G1	42	14	28	0.006
	G2 + G3	67	41	26	
Distant metastasis	Yes	42	36	6	0.000
	No	67	19	48	
Tumor size (cm)	≤ 10	62	34	28	0.336
	> 10	47	21	26	

### Cell culture and transfection

Standard ovarian cancer cell lines (SKOV3, IGROV1, A2780, and OVCAR3) and a normal human ovarian surface epithelial cell line (HOSE 6.3) were obtained from the American Type Culture Collection. Ovarian cancer cell lines were routinely cultured in DMEM (OVCAR-8 and SKOV-3) or RPMI 1640 (A2780 and IGROV-1), supplemented with 10 % fetal bovine serum (FBS; Gibco, Grand Island, NY, USA), 100 U/ml penicillin sodium, and 100 mg/ml streptomycin sulfate. HOSE 6.3 cells were maintained in MCDB Medium 199 (1:1, v/v) containing 10 % FBS [18]. All cells were cultured in a humidified atmosphere containing 5 % CO_2_ at 37 °C.

SKOV3 cells were transfected with siRNA (50 nM) targeting CCAT2 (si-CCAT2) or a scrambled negative control (si-NC) (GenePharma, China) using Lipofectamine 2000 (Invitrogen) according to the manufacturer’s protocol. The sequence selected for CCAT2 siRNA was 5’-AGGTGTAGCCAGAGTTAAT-3’ [13]. Cells were cultured for 48 h after transfection, and harvested to determine transfection efficiency by qRT-PCR.

### RNA extraction and qRT-PCR

Total RNA was extracted from ovarian cancer tissue samples, normal ovarian tissue specimens and cultured cells using TRIzol reagent (Invitrogen, Carlsbad, CA) following the manufacturer’s instructions. RNA concentrations and purity were measured spectrophotometrically at 260 and 280 nm. RNA was reverse transcribed to cDNA by using Reverse Transcription Kit (Takara) according to the manufacturer’s instructions. Real-time qPCR was performed with a SYBR RT-PCR kit (Takara) on an ABI 7500 Real-Time PCR System. GAPDH was used as an internal control. The following primers were used: GAPDH sense 5’-GCACCGTCAAGGCTGAGAAC-3’ and reverse 5’-TGGTGAAGACGCCAGTG GA-3’; CCAT2 sense 5’-CCCTGGTCAAATTGCTTAACCT-3’ and reverse 5’-TTATTCGTCCCTCTGTTTTATGGAT-3 [13]. Fold-changes of CCAT2 levels were derived by the 2^−ΔΔCt^ method, with GAPDH as a reference gene. Each test was performed in triplicate.

### MTT assay

SKOV3 cell proliferation was evaluated by the MTT (Sigma) assay according to the manufacturer’s instructions. SKOV3 cells transfected with either si-CCAT2 or si-NC for 48 h were reseeded into 96-well plates. Cell density was adjusted to 5 × 10^3^/well, for a final volume of 150 μl/well. After 24, 48, 72, and 96 h of incubation, MTT solution (20 μl/well) was added to the plates. The cells were further cultured for 4 h at 37 °C. Then, the medium was discarded, with 150 μl DMSO added for 15 min with shaking. Absorbance was measured at 490 nm on an enzyme-labeled analyzer. Three independent experiments were performed.

### Wound healing assay

Migration ability of cells was measured by a wound healing assay *in vitro*. Briefly, 2 × 10^5^ SKOV3 cells were seeded onto 6-well plates, with either si-CCAT2 or si-NC, and incubated in appropriate complete culture medium for 16 h in normoxic conditions at 37 °C. The monolayer was scratched and incubated in fresh medium for 24 h. The wound width was measured after 24 h. Three different locations were visualized and photographed under a phase-contrast inverted microscope (Leica, Solms, Germany).

### Transwell invasion assay

Cell invasion assay was performed using 24-well Transwell plates (8-μm pore size, Corning Life Sciences) coated with 1 mg/ml Matrigel (BD Sciences). SKOV3 cells transfected with either si-CCAT2 or si-NC were resuspended in serum-free medium at 1 × 10^5^ cells/ml, respectively. Each cell suspension was seeded in the upper chamber of the Transwell plate in 200 μl FBS-free medium; the lower chamber was filled with 500 μl 20 % FBS in medium. After 48 h of incubation at 37 °C with 5 % CO_2_, cells on the filter surface were fixed with methanol, stained with 0.1 % crystal violet, and photographed under a phase-contrast inverted microscope (Leica). Cells from at least five random microscopic fields (×100) were counted.

### Statistical analysis

Between-group analysis was carried out by independent *t*-test for continuous data; Chi-square test was used for categorical data. Overall survival (OS) and disease-free survival (DFS) curves were established by the Kaplan-Meier method, and analyzed by the log-rank test. A Cox proportional hazard model was constructed to evaluate the associations of CCAT2 expression with OS and DFS, respectively. Two-sided *P <* 0.05 was considered statistically significant. All statistical analyses were performed with the SPSS software version 17.0 (SPSS Inc., Chicago, IL, USA).

## Results

### CCAT2 gene expression is elevated in ovarian cancer tissues and cells

**Fig. 1:**
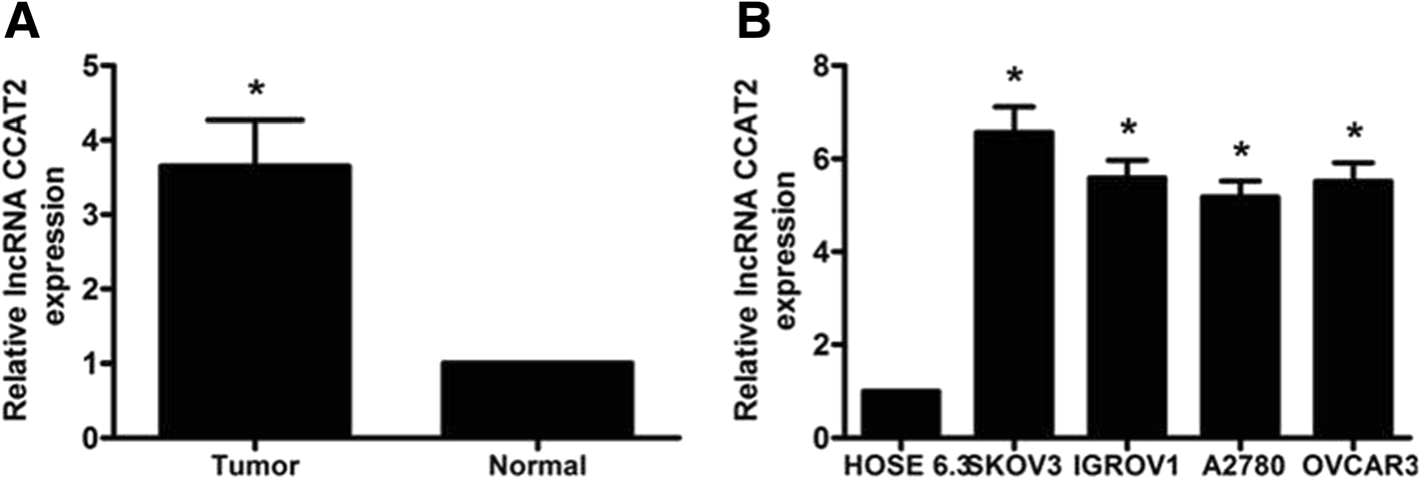
CCAT2 gene expression levels in ovarian cancer tissues and cells. Gene expression levels of the lncRNA CCAT2 were assessed by qRT-PCR, with GAPDH as an internal control. **a** CCAT2 expression levels in ovarian cancer samples were significantly higher than those in adjacent non-tumor tissues. **b** Higher expression levels of CCAT2 were detected in 4 ovarian cancer cell lines compared with values obtained for the normal human ovarian surface epithelial HOSE 6.3 cell line. Data are mean ± SD from triplicate experiments *P < 0.05

Using qRT-PCR, CCAT2 gene expression was assessed in 109 ovarian cancer tissue samples, 45 normal ovarian tissue specimens, and cultured ovarian cancer- or normal cells. Starkly increased CCAT2 gene expression levels were found in ovarian cancer tissue samples compared with normal ovarian tissue specimens (Fig. 1a, *P* < 0.01). In addition, CCAT2 was significantly up-regulated in four ovarian cancer cell lines (SKOV3, IGROV1, A2780, and OVCAR3) compared with normal human ovarian surface epithelial HOSE 6.3 cells (Fig. 1b, *P* < 0.01).

### Associations of CCAT2 expression with clinicopathological features in ovarian cancer patients

To further explore the role of CCAT2 in ovarian cancer, we next evaluated the associations its gene expression with several clinicopathological features. The 109 patients were divided into two groups by the median value of relative CCAT2 gene expression levels: high (n =55) and low (n =54) expression groups. As shown in Table 1, high CCAT2 level was not associated with patient age (*P* = 0.702), histological subtype (*P* = 0.918) or tumor size (*P* = 0.336); however it was significantly positively correlated with FIGO stage (*P* = 0.002), tumor grade (*P* = 0.006) and distant metastasis (*P* < 0.001). Taken together, these results supported the notion that CCAT2 up-regulation may be associated with tumor progression and development.

### High CCAT2 levels are correlated with poor prognosis in ovarian cancer patients

**Fig. 2:**
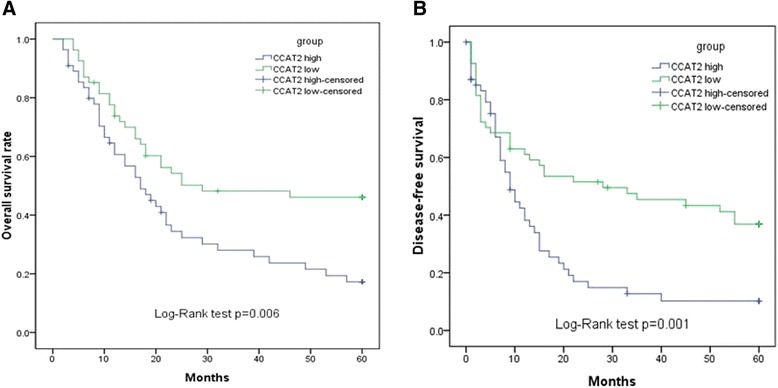
Kaplan-Meier survival curves for cervical cancer patients according to CCAT2 gene expression level. OS and DFS of patients with high vs. Low CCAT2 expression levels are shown. **a** OS of ovarian cancer patients with high CCAT2 expression was significantly poorer compared with rates found in patients with low CCAT2 levels (*P* < 0.05). **b** DFS of ovarian cancer patients with high CCAT2 expression was significantly poorer compared with rates in patients with low CCAT2 (*P* < 0.05)

To further explore the potential clinical value of high CCAT2 expression, the prognostic power of the lncRNA CCAT2 was determined for OS and DFS in 109 ovarian cancer patients, using the Kaplan-Meier method and log-rank test. Interestingly, high CCAT2 expression was correlated with shorter OS or DFS (*P* < 0.05, Fig. 2a and 2b). Univariate Cox proportional hazards regression model analysis showed that relative CCAT2 expression level, distant metastasis, tumor grade, and FIGO stage were correlated with overall survival rate in ovarian cancer patients (*P* < 0.05, Table 2). In addition, multivariate analysis revealed CCAT2 expression level, distant metastasis, tumor grade, and FIGO stage to be independent prognostic indicators for overall survival (*P* < 0.05, Table 2). These data suggested that the lncRNA CCAT2 could be a bona fide prognostic marker for ovarian cancer.

**Table 2:** Univariate and multivariate analyses of prognostic parameters in ovarian cancer patients by Cox regression analysis

Variable	Univariate analysis			Multivariate analysis		
	Risk ratio	95 % CI	*P-*value	Risk ratio	95 % CI	*P-*value
Age (years)	1.673	0.647–2.309	0. 614			
≥ 55 vs < 55						
Histologic grade	1.615	0.855–2.742	0.556			
Serous vs (Endometrioid + Mucinous + Clear cell + Others)						
Tumor size (cm)	1.017	0.722–2.209	0.658			
≤ 10 vs > 10						
Tumor grade	3.161	1.719–5.839	0.016	3.015	1.623–6.286	< 0.001
G1 vs G2 + G3						
Distant metastasis	3.957	1.892–7.413	<0.001	3.253	1.692–6.741	< 0.001
Yes vs No						
FIGO stage	2.419	1.401–5.811	0.005	2.724	1.412–5.509	0.003
I + II vs III + IV						
lncRNA CCAT2	3.227	1.638–6.279	0.001	2.938	1.526–5.873	< 0.001
High vs Low						

### Knockdown of the lncRNA CCAT2 inhibits cell proliferation, migration and invasion

**Fig. 3:**
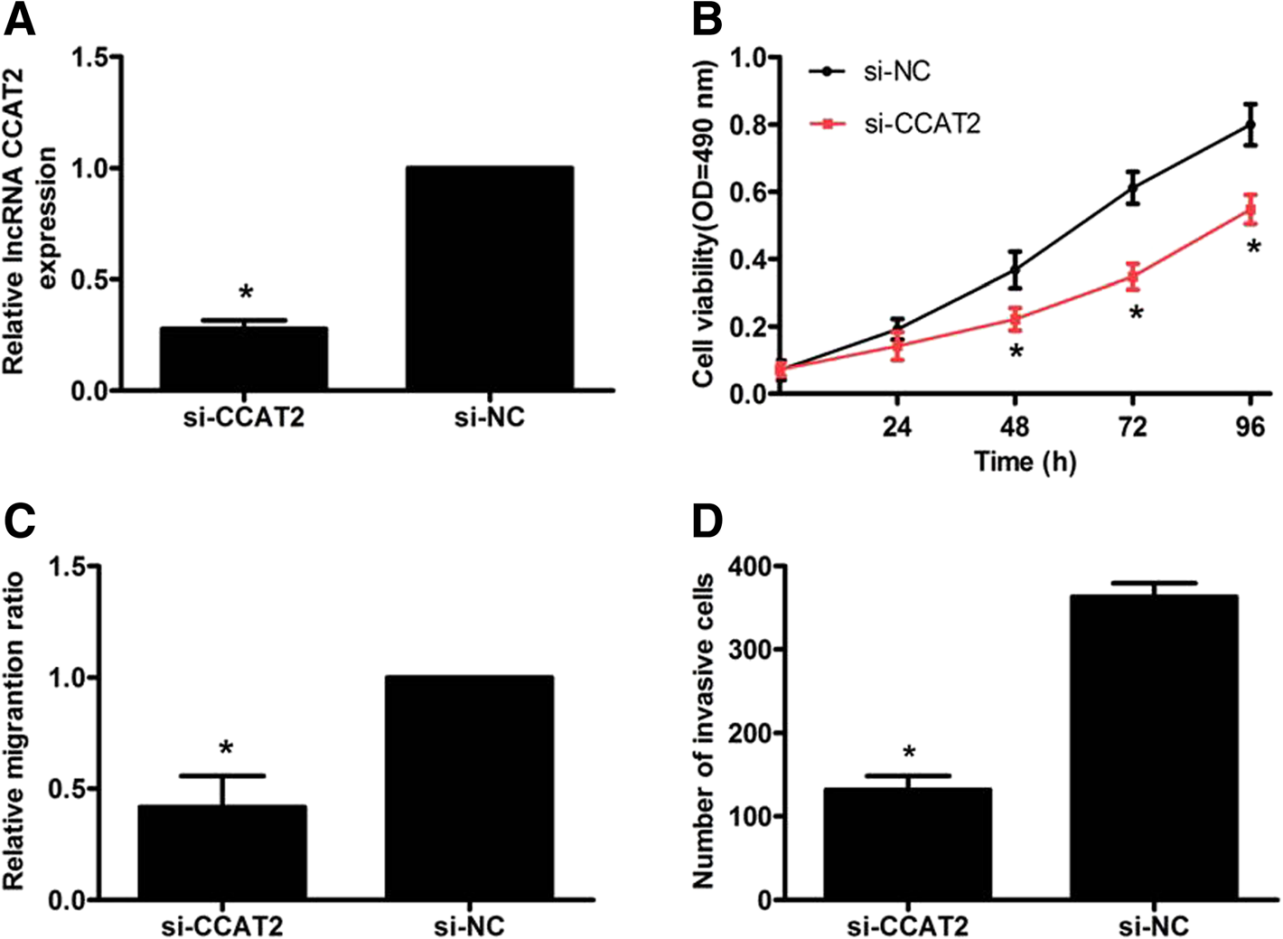
Knockdown of the lncRNA CCAT2 inhibits proliferation, migration and invasion in SKOV3 cells. **a** qRT-PCR revealed that CCAT2 was efficiently knocked down by treatment with si-CCAT2 in SKOV3 cells. **b** SKOV3 cells transfected with si-CCAT2 displayed significantly lower proliferation ability compared with those transfected with si-NC. **c** SKOV3 cells transfected with si-CCAT2 showed markedly lower migration ability compared with those transfected with si-NC. **d** SKOV3 cells transfected with si-CCAT2 displayed significantly lower invasion ability compared with those transfected with si-NC. Data are mean ± SD from triplicate experiments. *P < 0.05

In order to explore the functional role of CCAT2 in ovarian cancer tumorigenesis, SKOV3 cells were transfected with si-CCAT2 or si-NC. 48 h after transfection, qRT-PCR was performed to assess CCAT2 gene expression. As shown in Fig. 3a, cells transfected with si-CCAT2 had significantly decreased CCAT2 mRNA levels compared with the si-NC group (*P* < 0.05).

Next, cell viability was assessed by MTT assay. As shown in Fig. 3b, SKOV3 cell transfection with si-CCAT2 resulted in significantly decreased viability compared with the si-NC group. Furthermore, the role of the lncRNA CCAT2 in cell migration and invasion was assessed. In wound healing assay, the migration ability of SKOV3 cells transfected with si-CCAT2 was significantly decreased compared with that of the si-NC group (*P* < 0.05, Fig. 3c). In addition, Transwell invasion assay revealed reduced invasion capacity for SKOV3 cells transfected with si-CCAT2 in comparison with the si-NC group (*P* < 0.05, Fig. 3d). These findings demonstrated that CCAT2 silencing could inhibit cell proliferation, migration and invasion *in vitro*.

## Discussion

Ovarian cancer, a gynecological malignancy, has been a leading cause of death among women for the past decades. The dismal prognosis of ovarian cancer is due to few symptoms in early-stage; patients are generally diagnosed with advanced-stage tumors, which are less susceptible to existing treatments [19]. Therefore, it is important to explore the molecular events in ovarian cancer, which would provide insights for improved diagnosis and prognosis of this deadly disease, thus improving clinical strategies and outcomes.

It was previously demonstrated that the majority of RNA transcripts in mammalian cells do not encode proteins; indeed, protein-coding genes account for only 2 % of the total genome, whereas much of the human genome, far more than expected, is transcribed into noncoding RNAs [20]. Among these, lncRNAs have been confirmed to be important biological RNAs, regulating gene expression at the transcription, epigenetic, or translation level [21]. Increasing evidence suggests that lncRNAs are emerging and important regulators of several biological processes, playing oncogenic or tumor suppressor roles in tumorigenesis [22, 23]. The lncRNA CCAT2 is an imprinted and maternally expressed gene, which promotes the progression of lung [14] and gastric [16] cancers. However, CCAT2 expression and prognostic role in ovarian cancer remain unclear.

In the present study, CCAT2 gene expression levels were significantly higher in ovarian cancer tissue samples and cell lines. To assess whether CCAT2 expression can serve as a novel prognostic marker, potential associations of CCAT2 expression with clinicopathological factors were examined. Interestingly, patients with high CCAT2 gene expression levels showed poor prognostic parameters, including FIGO stage and distant metastasis.

In addition, patients with high CCAT2 expression levels had a significantly poorer prognosis compared with the low expression group. As shown above, shorter overall- and disease-free survival times were observed in the high CCAT2 expression group compared with values obtained for the low expression group. Moreover, multivariate Cox analysis indicated that high CCAT2 expression was an independent predictor of ovarian cancer. Taken together, these findings demonstrated for the first time the clinical significance of the lncRNA CCAT2 in ovarian cancer.

Zhang et al [24] demonstrated that the lncRNA CCAT2 is upregulated in esophageal squamous cell carcinoma (ESCC) tissues, with positive correlations between CCAT2 level and lymph node metastasis, TNM stage, and the number of positive lymph nodes, respectively. In addition, previous findings demonstrated that CCAT2 is upregulated in breast tumors; indeed, CCAT2 silencing decreases cell proliferation and invasion *in vitro* through the Wnt/β-catenin signaling pathway [25]. Thus, further experiments are needed to identify the biological processes regulated by CCAT2 in ovarian cancer. We next silenced CCAT2 in SKOV3 cells *in vitro*. As shown above, knockdown of the lncRNA CCAT2 inhibited cell proliferation, migration and invasion, further highlighting the role of CCAT2 in cell biology and oncogenesis of ovarian cancer cells.

## Conclusion

In summary, this is the first report showing that lncRNA CCAT2 expression is significantly high in ovarian cancer tissues and cell lines. In addition, higher CCAT2 gene expression level was associated with poor prognosis in ovarian cancer patients. Moreover, CCAT2 silencing in ovarian cancer cells markedly suppressed cell proliferation, migration, and invasion. Taken together, these findings suggest a potentially important role for CCAT2, which could serve as a potential biomarker and therapeutic target in ovarian cancer.

## Abbreviations

CCAT2, Colon cancer-associated transcript 2; DFS, Disease-free survival; DMSO, Dimethyl sulfoxide; ESCC, Esophageal squamous cell carcinoma; FBS, Fetal bovine serum; FIGO, International Federation of Gynecology and Obstetrics; GAPDH, Glyceraldehyde-3-phosphate dehydrogenase; lncRNAs, Long non-coding RNAs; MTT, 3-(4,5)-dimethylthiahiazo (−z-y1)-3,5-diphenytetrazoliumromide; OS, Overall survival; qRT-PCR, Quantitative real-time PCR; si-CCAT2, siRNAs targeting CCAT2; si-NC, Scrambled negative controls; siRNA, Small interfering RNA; TNM, Tumor node- metastasis.
